# Contrasting sensitivity of soil bacterial and fungal community composition to one year of water limitation in Scots pine mesocosms

**DOI:** 10.1093/femsec/fiad051

**Published:** 2023-05-15

**Authors:** Astrid C H Jaeger, Martin Hartmann, Johan Six, Emily F Solly

**Affiliations:** Department of Environmental Systems Science, Sustainable Agroecosystems Group, Zurich, ETH Zurich, Switzerland; Department of Environmental Systems Science, Sustainable Agroecosystems Group, Zurich, ETH Zurich, Switzerland; Department of Environmental Systems Science, Sustainable Agroecosystems Group, Zurich, ETH Zurich, Switzerland; Department of Environmental Systems Science, Sustainable Agroecosystems Group, Zurich, ETH Zurich, Switzerland

**Keywords:** DNA metabarcoding, mesocosm experiment, Scots Pine (Pinus Sylvestris L), seasonality, soil microbiome, water limitation

## Abstract

The soil microbiome is crucial for regulating biogeochemical processes and can, thus, strongly influence tree health, especially under stress conditions. However, little is known about the effect of prolonged water deficit on soil microbial communities during the development of saplings. We assessed the response of prokaryotic and fungal communities to different levels of experimental water limitation in mesocosms with Scots pine saplings. We combined analyses of physicochemical soil properties and tree growth with DNA metabarcoding of soil microbial communities throughout four seasons. Seasonal changes in soil temperature and soil water content and a decreasing soil pH strongly influenced the composition of microbial communities but not their total abundance. Contrasting levels of soil water contents gradually altered the soil microbial community structure over the four seasons. Results indicated that prokaryotic communities were less resistant to water limitation than fungal communities. Water limitation promoted the proliferation of desiccation tolerant, oligotrophic taxa. Moreover, water limitation and an associated increase in soil C/N ratio induced a shift in the potential lifestyle of taxa from symbiotic to saprotrophic. Overall, water limitation appeared to alter soil microbial communities involved in nutrient cycling, pointing to potential consequences for forest health affected by prolonged episodes of drought.

## Teaser

Despite the influence of the sampling time point, water limitation altered soil microbial community structures in Scots pine mesocosms, but fungal taxa were less sensitive to reduced soil water contents.

## Introduction

Precipitation projections in future climate scenarios are variable and uncertain; nevertheless, general circulation models project a consistent increase in air temperature and, thus, evapotranspiration in Europe, resulting in decreased soil moisture in many regions (Vogel et al. 2018, van der Linden et al. 2019). In general, the reduction of soil moisture will force soil microbes to either avoid or tolerate water-limited conditions while striving for nutrient and energy sources that become spatially less available (Manzoni et al. 2014).

Soil water availability controls soil microbial community dynamics in three fundamental ways: as a resource, solvent, and transport medium (Tecon and Or 2017). All of these are important for regulating the function of the soil microbiome (Schimel 2018). Therefore, a declining soil water content can create environmental stress for microbes (Schimel et al. 2007). Moreover, water scarcity can directly affect the soil microbiome by creating osmotic stress, leading to microbial death and cell lysis (Csonka 1989, Turner et al. 2003). However, it is expected that some taxa evolve drought resistance, e.g. by accumulating osmolytes (Schimel et al. 2007, Warren 2014), producing exopolysaccharides (Roberson and Firestone 1992), forming thick cell walls (Potts 1994), or entering a dormant state (Lennon and Jones 2011).

Although bacteria endure desiccation by shifting to dormancy, producing cysts, or inhabiting small soil pores (Schimel et al. 2007, Lennon and Jones 2011), fungi are often considered to be more tolerant to water limitation than bacteria (Schimel et al. 2007, Strickland and Rousk 2010, Manzoni et al. 2012). Important assets of fungi under dry conditions, include osmolytes, thick cell walls, and melanin (Schimel et al. 2007). Moreover, the higher tolerance is mainly attributed to the ability of fungi to create large hyphal networks that maintain nutrient and water translocation over long distances and allow the scavenging of small soil pores filled with water (Allen 2007, Joergensen and Wichern 2008). Soil ecosystems dominated by fungi are, as such, assumed to be more resistant to desiccation than soils dominated by bacteria (Yuste et al. 2011, De Vries et al. 2012). Furthermore, it has been proposed that fungi have lower nutrient requirements than bacteria (Güsewell and Gessner 2009, Strickland and Rousk 2010), and that fungi can utilize a broader range of nutrient resources (Sterner and Elser 2003).

In addition to the mentioned direct physical effects, altered water availability may impact the soil microbiome indirectly through changes in vegetation or substrate supply (Nielsen and Ball 2015). Plants undergo a set of physiological reactions in response to water deficit. Adaptations to lower soil water contents result inter alia in changes in the amount and chemical quality of plant litter input to the soil (Cromer et al. 1984) and altered root growth and exudation profiles (Brunner et al. 2015, Hasibeder et al. 2015). For example, the quality of litter input decreases under drought through enhanced production of recalcitrant structural compounds by plants (Pugnaire et al. 2019). These changes consecutively slow down mineralization rates and nutrient release, further affecting the functional structure and activity of the microbial communities (Bolton et al. 1992, Grayston et al. 1998). In dry soils, an increased abundance of microbial genes involved in the degradation of complex plant polysaccharides has been observed, while the quantity of microbial genes targeting less complex oligosaccharides of fresh organic matter inputs often decreases (Bouskill et al. 2013, Martiny et al. 2017). Therefore, it is proposed that drought-related decreasing plant vitality causes a community succession toward taxa capable of degrading complex structures of dead plant material enhancing saprotrophic taxa (Voriskova and Baldrian 2013, Kielak et al. 2016b, Baldrian 2017, Herzog et al. 2019). Concurrently, symbionts such as ectomycorrhizal fungi (EcM) and certain diazotrophic bacteria depend on their host plants (Nehls 2008, Carvalho et al. 2014, Mercado-Blanco et al. 2018). Therefore, the abundance and richness of symbiotic taxa are expected to decline with changing environmental conditions due to impaired interaction with the host (Churchland and Grayston 2014, Knoth et al. 2014).

Impaired tree growth and physiology have been documented in several areas of the European Central Alps, as in Switzerland, Italy, and Austria (Vertui and Tagliaferro 1998, Rebetez and Dobbertin 2004, Rigling et al. 2018), with Scots pine (*Pinus Sylvestris* L.) being one of the dominant tree species of inner-Alpine forests (Leuschner and Ellenberg 2017). Although average annual precipitation has remained constant over the last decades, there is evidence that climate warming has increased evapotranspiration rates and that water has become a main factor affecting the vitality and stress resilience of trees (Rigling et al. 2013).

Previous findings from a long-term irrigation study conducted in a xeric mature forest stand (Hartmann et al. 2017) indicated that water-limiting conditions favor oligotrophic, metabolically versatile, and drought-tolerant taxa. There is, however, little information available on potential changes in microbial communities under different levels of water limitation over the whole growing season of trees. To assess whether observations from field studies can be confirmed under controlled experimental conditions during the growing season, we conducted a one-year mesocosm experiment with Scots pine saplings and natural soil from a xeric mature forest stand (Herzog et al. 2014, 2019).

The main goal of this study was to assess whether and how soil prokaryotic and fungal communities respond to different levels of experimental water limitation in Scots pine mesocosms. Moreover, we were interested in understanding if variations in soil microbial communities are related to alterations in tree growth and soil physicochemical properties. We hypothesized that severe water limitation and altered resource availabilities would cause community succession toward an enhanced abundance of saprotrophic taxa capable of degrading complex organic compounds of dead plant material. At the same time, we expected that water limitation would decrease symbiotic taxa through changes in physicochemical soil properties. Moreover, we presumed that desiccation-tolerant taxa and oligotrophic bacteria would increase under water stress. Finally, we hypothesized that the composition of soil fungal communities would be more resistant to water limitation than prokaryotic communities.

## Materials and Methods

### Experimental set-up

For the experiment, natural forest soil was collected in a xeric forest in the upper Rhone Valley (Pfyn forest, Canton Valais, Switzerland, 46°18′16.1′N, 7°36′44.8′E, and 600 m a.s.l.) in November 2018. The soil (Pararendzina, developed on an alluvial fan) was collected at the forest margins of the Pfyn Nature Park, below the forest canopy. For the soil collection, an excavator was used to remove the 3–6 cm deep organic horizon (Oe horizon), and ∼4 t of soil were taken from the upper ∼35 cm of the mineral soil (sand/silt/clay %: 49/43/8, skeletal material: 20%–50%, and bulk density: 1.3 g cm^−3^). The soil was transported in soil bulk bags to the greenhouse facility at the Research Station for Plant Sciences Lindau (ETH Zurich, Switzerland) and stored outside on wooden pallets, covered for protection against rain. The soil was homogenized with a steel riffle splitter, and large stones were removed. In February 2019, part of the soil was used for the initial planting of 2-year-old Scots pine saplings (*Pinus Sylvestris* L.) in pots (6 L volume). The Scots pine had been growing from seeds in a common potting substrate (seed origin: Leuk, Switzerland, 980–1250 m a.s.l.) at the tree nursery of the Swiss Federal Research Institute for Forest, Snow and Landscape Research (WSL, Birmensdorf, Switzerland). The purpose of this initial planting was to acclimate the saplings to the forest soil for the duration of one growing season.

**Figure 1.**
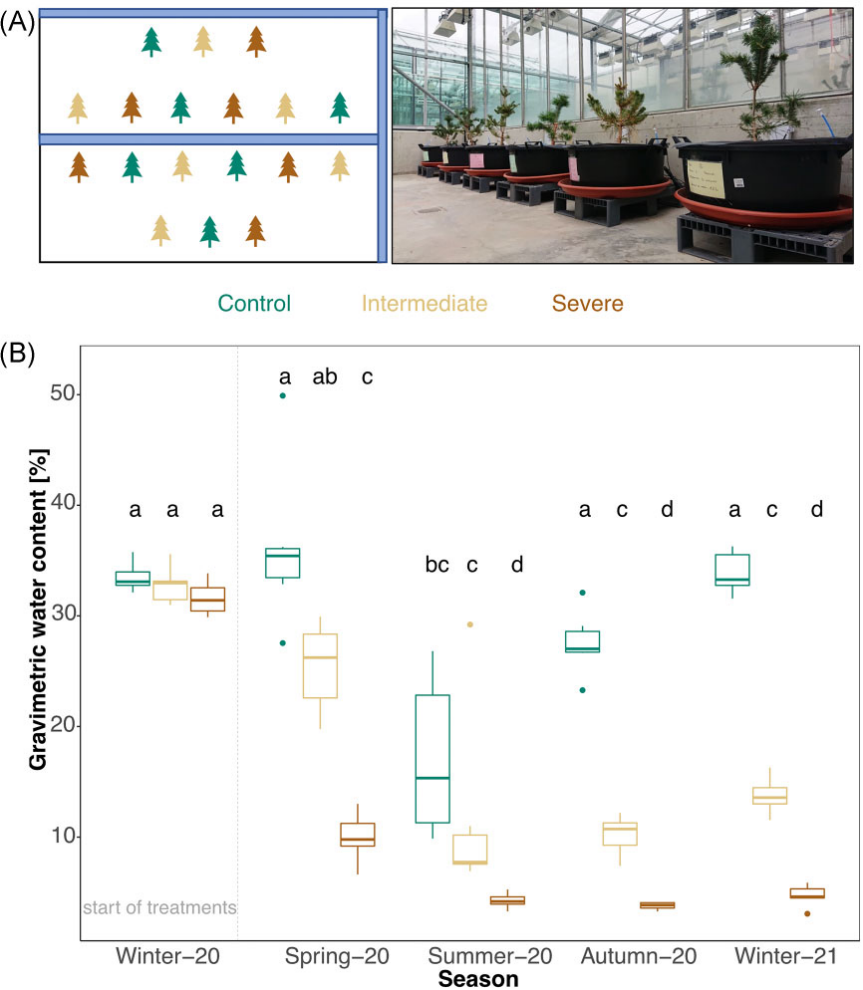
Experimental set-up of the Scots pine soil mesocosms in the greenhouse. (A) Set-up of the experiment with a total of 18 mesocosms treated with three different levels of irrigation (control, intermediate, and severe). (B) Gravimetric water content in % for each treatment (*n* = 6) at all sampling time points (seasons). Small letters indicate significant differences between groups based on estimated marginal means of linear mixed effect models.

In September 2019, 18 of the Scot pine saplings (which had by then experienced three growing seasons) were individually transplanted in pots with a size of 32 cm height × 69 cm diameter (100 L volume) at the greenhouse facility. During the transplantation of each Scots pine, the pots were filled with a 2–3 cm layer of stones (10–15 kg) and 20 cm of forest soil (100–110 kg). For three months, the plant-soil systems (subsequently referred to as “mesocosms”) were watered twice per week with 2 L of local rainwater reaching a volumetric water content (VWC) of ∼30% (close to field capacity, which was ∼35% for the soil, with a pF of 1.8). In January 2020, the mesocosms were assigned to three different irrigation treatments in a randomized design to minimize spatial effects (i.e. variability in shading). The three levels of irrigation were: sufficient water supply (control; 30% VWC, *n* = 6), decreased amount of water (intermediate; 40% reduction in VWC of control, *n* = 6), and water stress (severe; 75% reduction in VWC of control, *n* = 6) (Fig. 1). The intermediate treatment represents the maximum forecasted deviation of precipitation from the normal under a future climate in Southern Switzerland (period: 2081–2100) compared to 1981–2010 without climate change mitigation (CH2018–Climate Scenarios for Switzerland 2018). The severe treatment was chosen to maximize the effect of water stress. However, the soil water content was kept at a level, at which the saplings received a minimum of water not to suffer from permanent damage and maintain vitality. The greenhouse temperatures were regulated to simulate seasonal changes experimentally (Supplementary Table 1) and constantly controlled, together with the humidity, which was kept around 50%–70% throughout the seasons (Supplementary Table 1). The mesocosms were equipped with soil sensors continuously measuring VWC, soil water potential, and soil temperature (Teros 11, Teros 21, Meter Group, Pullman, WA, USA).

### Scots pine structure and photosynthetic capacity

The structure of the Scots pine was monitored by measuring height and diameter every month as a proxy of aboveground plant productivity. The height was measured, including the buds, and the seasonal increment was calculated as tree growth. The diameter was measured at two angles, and the mean was taken. Here the seasonal increment was calculated as radial growth. The needle fall was collected each season on a PE net (mesh size 3 × 3 mm) placed above the pot. The collected needle litter was weighed, dried for one week at 40°C, and redistributed on the soil surface according to the average needle fall for each of the three irrigation treatments. The net photosynthetic assimilation was measured every season to assess the maximum rate at which leaves are able to fix carbon during photosynthesis as a proxy of plant vitality (Johnstone et al. 2013). Therefore, 25 south exposed needles were enclosed in the 2 × 3 cm chamber of the LI-COR LI-6400 (LI-COR Biosciences, Lincoln, N.E., USA), and net photosynthetic assimilation (A_net_) was measured under 400 μmole mol^−1^ CO_2_, 1000 PAR, local humidity and temperature, and a stomatal ratio of 1.

### Soil sampling

Bulk soil sampling was performed at different stages of plant growth and temperature settings —September 2019, January 2020, Mai 2020, July 2020, October 2020, and January 2021— in all pots (*n* = 18) with a slide-hammer corer (30 cm length, 5.5 cm diameter) to a depth of 25 cm. In the following text, these sampling time points will be termed “seasons” to account for differences in plant growth and greenhouse temperatures and referred to as “Autumn-19,” “Winter-20,” “Spring-20,” “Summer-20,” “Autumn-20,” “Winter-21”. After sampling, the soil samples were kept cooled, transported to the lab, and immediately stored at 4°C.

### Physicochemical soil properties

The day after each sampling, the fresh soil samples were sieved to 4 mm, and large organic residues were sorted out manually. Fresh soil samples were used to analyse gravimetric water content (GWC, 10 g of soil) and inorganic nitrogen (NH_4_^+^, NO_3_^−^, 10 g of soil) and to perform DNA extraction (0.250 g). Soil samples for molecular analysis were frozen at −20°C until further processing. The rest of the soil samples were dried at 40°C to constant weight and sieved to 2 mm for measurements of soil pH, total carbon (TC), inorganic carbon (IC), organic carbon concentration (C_org_), and total nitrogen concentration (TN).

Soil GWC was assessed by weighing a subsample of 10 g of soil before and after drying at 105°C for at least 48 h. Ammonium (NH_4_^+^) and nitrate (NO_3_^−^) concentrations of the soils were determined by extraction of 10 g soil with 50 ml 2 M KCl solution in a 1:5 soil solution ratio and shaken for 1 h at 180 r/m. Afterward, the soil–KCl solution was filtered with a 150 nm ashless filter paper (Whatman No. 42), and the clear solution was stored at −20°C until further analyses. NH_4_^+^ and NO_3_^−^ concentrations were determined colorimetrical with a spectrophotometer v-1200 (VWR, Radnor, PA, USA) following Forster (1995) for NH_4_^+^ and Doane and Horwáth (2003) for NO_3_^−^. Soil pH was measured in a 1:2.5 solution containing 10  g of dried soil and 25 ml of 0.01 M CaCl_2_ solution. Samples were shaken horizontally at 180 r/m for 1 h and stored overnight to allow sedimentation before measurement with a pH-meter (VWR, Radnor, PA, USA). TC and nitrogen concentrations were determined with an elemental analyser LECO 628 (LECO, St. Joseph, MI, USA). IC was measured with the pressure-calcimeter method following Sherrod et al. (2002). The C_org_ was calculated as follows: C_org_ = TC–IC and used to determine C_org_/TN, which will be subsequently referred to as C:N ratio.

### Microbial gene abundance

DNA extraction from 0.250 g fresh soil was performed with the DNeasy PowerSoil Pro Kit (Qiagen, Hilden, Germany) according to the manufacturer’s instructions using the QIACube System (Qiagen, Hilden, Germany). Quality and quantity of extracted DNA were measured via UV/VIS spectrophotometry with the QIAxpert System (Qiagen, Hilden, Germany). The abundance of taxonomic groups —bacteria and archaea, hereafter termed as prokaryotes, and fungi— was assessed using a SYBR^®^ Green-based qPCR approach. Potential amplification inhibition induced by unintentional co-extraction of contaminants was tested across all samples by spiking pGEM-T plasmid (GenBank^®^ Accession No. X65308; Promega, Madison, WI, USA) into the soil DNA at equimolar concentrations in all samples and amplifying a region on the plasmid using specific primers SP6 and T7 (Microsynth, Balgach, Switzerland). The qPCR standards were produced from purified PCR products obtained by pooling DNA from randomly selected samples. For the standard curves, the concentrations ranging from 10^−2^ to 10^−8^ ng of DNA template were utilized for all reactions. The selected primers 515F (Parada et al. 2016) and 806R (Frey et al. 2016) (Microsynth, Balgach, Switzerland) were targeting the 16S rRNA gene (V4 region). The primers FF390 (nu-SSU-1334–5′) (Microsynth, Balgach, Switzerland) and FR1 (nu-SSU-1648–3′) (Microsynth, Balgach, Switzerland) (Vainio and Hantula 2000), were chosen to target the 18S rRNA gene (V7-V8 region). All qPCR reactions were performed in a final volume of 25 µL containing a final concentration of 0.75 µM of primer, 1x Sso Advanced™ Universal SYBR^®^ Green Supermix (Bio-Rad Laboratories, Hercules, CA, USA), and 10 ng of DNA template. The conditions for the qPCR assays were as follows: 3 min for enzyme activation at 98°C, followed by 15 s for denaturation at 95°C, 30 s primer hybridization at 52°C for 16S and 50°C for 18S, respectively, and 30 s for elongation at 72°C, for 35 cycles. To verify the amplification specificity, melting curves were generated by increasing the temperature from 75°C to 95°C by 0.5°C every 5 s at the end of the amplification cycles. All qPCRs were performed in technical triplicates in a thermocycler CFX96 Touch Real-Time System (Bio-Rad Laboratories, Hercules, CA, USA), and the results were documented and analysed using the CFX Maestro software (Bio-Rad Laboratories, Hercules, CA, USA). To check for between run differences, eight standard samples were repeated in every plate and compared. The qPCR efficiency was between 90% and 100% with an R^2^ > 0.99 for all runs.

### Microbial community structure

Changes in prokaryotic and fungal community structure were investigated with DNA metabarcoding of ribosomal marker genes. PCR amplification of the prokaryotic 16S rRNA gene (V3–V4 region) was performed with the primers 341F and 806R (Frey et al. 2016). Primers ITS3ngs and ITS4ngs were used to amplify the fungal ITS2 region of the rrn operon (Tedersoo and Lindahl 2016). PCR reactions contained 1X of GoTaq^®^ G2 Hot start Master Mix from Promega (Promega, Madison, WI, USA), 0.4 µM of the primers, and 40 ng of DNA template in a final volume of 25 µL. Cycling conditions for the PCR reactions consisted of a polymerase activation step at 95°C for 5 min, followed by denaturation at 95°C for 40 s, primer hybridization at 58°C, respectively, at 55°C for fungi for 40 s, and elongation at 72°C for 1 min, repeated in 30 cycles. PCR amplification was carried out in technical triplicates, and triplicates were pooled prior to sequencing. Pooled PCR products were sent to the Functional Genomics Center Zurich (FGCZ, Zurich, Switzerland) for indexing PCR. Indexed PCR products were purified, quantified, and pooled in equimolar ratios. Presequencing on the Illumina MiniSeq platform (Illumina, San Diego, CA, USA) was performed to inform library repooling in order to achieve optimal read count distributions across all samples. Final sequencing was executed on the Illumina MiSeq platform (Illumina, San Diego, CA, USA) using the v3 chemistry for PE300 reads.

### Bioinformatics

Sequencing data were processed using a customized bioinformatics pipeline largely based on VSEARCH v2.21.1 (Rognes et al. 2016). Primers used in PCR were trimmed with CUTADAPT v3.4 (Martin 2011), allowing for 1 mismatch. Bowtie2 v2.4.5 (Langmead and Salzberg 2012) was used to filter for PhiX contamination by aligning the reads against the PhiX genome (accession NC_001422.1). The *fastq_mergepairs* function in VSEARCH was used to merge trimmed paired-end reads, and the *fastq_filter* function was applied for quality filtering with a maximum expected error of 1 (Edgar and Flyvbjerg 2015). Sequences were dereplicated using the *derep_fulllength* function in VSEARCH and delineated into amplicon sequence variants (ASVs) applying the UNOISE algorithm (Edgar 2016a) of VSEARCH with an alpha of 2 and a minsize of 8. The UCHIME2 algorithm (Edgar 2016c) implemented as the *uchime3_denovo* function in VSEARCH was used to identify and remove potentially chimeric ASV sequences. Remaining ASV sequences were tested for the presence of ribosomal signatures using Metaxa2 v.2.2.3 (Bengtsson-Palme et al. 2015) for the 16S rRNA gene and ITSx v.1.1.3 (Bengtsson-Palme et al. 2013) for the ITS2 sequences, and non-matching sequences were discarded. The final ASV table was generated by mapping the quality-filtered reads of each sample against the verified ASV sequences executing the *usearch_global* algorithm implemented in VSEARCH with the following settings: maxhits 1, maxrejects 100, maxaccepts 0, and a minimum identity of 97%. Verified ASV sequences were taxonomically classified by running the SINTAX algorithm (Edgar 2016b) of VSEARCH against the SILVA v.138 database (Quast et al. 2013) for the 16S rRNA gene sequences and against the UNITE v.8.0 database (Abarenkov et al. 2010, Nilsson et al. 2019) for the ITS2 sequences, using a bootstrap cutoff of 0.8. ASVs not assigned at the domain level of bacteria, archaea, or fungi, as well as ASVs assigned to organelle structures (chloroplasts and mitochondria), were removed from the ASV table. The raw sequences were deposited in the European Nucleotide Archive (ENA) under the accession number PRJEB53192.

### Statistical analyses

All statistical analyses were computed in R Version 4.1.3 (R Core Team 2022) using RStudio Version 2022.02.1+461 (RStudio Team 2022). For all tests, a *P-value* <.05 was considered significant unless mentioned otherwise. To test the effect of the treatment and the sampling time point (season) on the investigated soil parameters (GWC, pH, C:N ratio, C_org_, TN, NH_4_^+^, NO_3_^−^), plant parameters (height, diameter, needle litter, and A_net_) and estimated copies of the 16S rRNA gene and the 18S rRNA gene, the data were fitted to linear mixed effect models. Here, the *lme* function of the package *nlme* v.3.1–157 (Pinheiro et al. 2022) was applied using the restricted maximum likelihood method “*REML*” (Meyer 1989). Whereas, irrigation treatment (control, intermediate, severe, *n* = 6) and sampling time point (Winter-20, Spring-20, Summer-20, Autumn-20, Winter-21) were used as factor variables with interaction. The greenhouse (greenhouses 1 and 2) and the pot number (pots 1–18) were assumed to be a nested random effect. When visual inspection of diagnostic residual plots revealed that data deviated from the assumption of homoscedasticity or normality, the data were transformed using *log* (for TN), *log_10_* (for GWC, NO_3_^−^, 16S gene copies), or *sqr*t (C:N ratio, NH_4_^+^). The data were back-transformed for further tests using the *ref_grid* function of the package *emmeans* v.1.7.3 (Lenth et al. 2022). To test the effect of the treatment and the sampling time point (season), pairwise comparisons were estimated by using marginal means with the *emmeans* function of the same package while adjusting for multiple comparisons with the *Sidak* method. To identify and display significant differences between groups, the function *cld* of the package *multcomp* v.1.4–18 (Hothorn et al. 2022) was applied.

Sequencing depth was investigated using barplots and rarefaction curves with the *rarecurve* function in the *vegan* package v.2.6–2 (Oksanen et al. 2022) ( Supplementary Figure 2). To account for differences in sequencing depth, changes in α-diversity (observed richness, Pielou’s evenness, and Shannon diversity) and β-diversity (Bray–Curtis dissimilarity) were calculated from 100-fold iteratively subsampled and square-root transformed ASV count tables (Martiny et al. 2017, Hemkemeyer et al. 2019). Here, the functions *rrarefy, specnumber, diversity*, and *vegdist* in *vegan* were applied. The effect of treatment and sampling time point (season) on α- and β-diversity were assessed using univariate or multivariate permutational analysis of variance PERMANOVA (Anderson 2001) and PERMDISP (Anderson 2006) with 999 permutations, as implemented in the *adonis2* and *betadisper* functions of *vegan*. Pairwise comparisons between factor levels were performed using the *pairwise.perm.manova* function from the package *RVAideMemoire* v.0.9–81-2 (Hervé 2022). Differences in β-diversity were assessed by unconstrained ordination using principal coordinate analysis (PCO) (Gower 2015) with the *cmdscale* function. Constrained ordination was performed using canonical analysis of principal coordinates (CAP) (Anderson and Willis 2003) implemented as the *CAPdiscrim* function of the B*iodiversityR* package v.2.14–1 (Kindt 2022), with 999 permutations, setting the factors treatment and sampling time point (season) as constraining factors. Here, the CAP reclassification success rate provides a quantitative estimation of the degree of discrimination between treatment groups. The effects of measured soil physiochemical properties and plant parameters on microbial communities were obtained using the PERMANOVA test. Additionally, factors labeled as significant in the PERMANOVA test were further used as a constraining factor in building a parsimonious model executing the function *ordistep* in *vegan*, and the significant factors were displayed by distance-based redundancy analysis (db-RDA) (Legendre and Andersson 1999) using the *dbrda* function in *vegan*. The response of individual taxonomic groups from phylum to genus level toward water limitation was assessed using univariate PERMANOVA based on Euclidean distances via the *adonis2* function with 9999 permutations on aggregated data at each taxonomic level, i.e. summing up the read counts of ASVs assigned to the same taxonomic group (Supplementary data). To adjust for multiple testing, q-values (Storey and Tibshirani 2003) were calculated using the *qvalue* function of the R package *qvalue* v.2.24.0 (Storey et al. 2022) and q-values < .05 considered significant, and q-values *< .1* as marginally significant. The data were z-transformed to visualize changes in relative abundance under each treatment, and the relative change in abundance compared to the control treatment was calculated.

A correlation-based indicator species analysis (De Cáceres and Legendre 2009, De Cáceres et al. 2010) was performed on significant ASVs from the PERMANOVA analysis to determine the association strength of each ASV with a sampling time point (season) and treatment or a combination, therefore. The function *multipatt* of the package *indicspecies* v.1.7.12 (De Cáceres et al. 2022) with a max.order of 10 was used. *P-value* adjustments for multiple comparisons were performed using the false discovery rate correction according to Storey (2002), and associations were considered significant at *q-value* < .05. Bipartite association networks were created using the software Cytoscape v.3.9.1 (Shannon et al. 2003). The bipartite networks were generated following Hartmann et al. (2015) by using the sampling time point (season) and treatment combination as source nodes and the ASVs as target nodes, with edges corresponding to positive associations of ASVs with sampling time point (season) and treatment combinations as obtained from the indicator species analysis Networks split by phyla were constructed using the Allegro Fruchtermann-Reingold algorithm (Fruchterman and Reingold 1991) with edges weighted according to the association strength.

For inferences about the potential lifestyle of the taxa, literature research was completed in conjunction with Faprotax v1.2.4 (Louca et al. 2016) and FUNGuild v.1.0 (Nguyen et al. 2016) for prokaryotes and fungi, respectively.

## Results

### GWC in soils

During the first four months of the experiment, the GWC of the soils was kept at ca. 30% in all mesocosms. Therefore, the GWC of the soils remained similar during the Winter-20 season (Fig. 1). Following the start of the three irrigation treatments (control, intermediate water limitation, and severe water limitation), the GWC of the soils consistently differed among the treatments throughout all samplings (*P* <.0001, Table 1). Throughout the sampling time points (season), the soils of the control mesocosms had gravimetric water contents ranging between 17% and 36%, whereas, the variability within the treatment group was highest in Summer-20, ranging between 10% and 28%. Soils of the mesocosm treated with intermediate and severe water limitation had gravimetric water contents ranging from 10% to 25% and 4% to 9%, respectively (Fig.   1). The GWC of the mesocosms exposed to the three treatments significantly varied across sampling time points (season, *P* <.0001, Table   1). The highest water content values were measured in Spring-20 for all treatments, and the lowest values in Summer-20 (Fig. 1).

**Table 1.** Effects of irrigation treatment and sampling time point (season) on different soil properties and tree parameters.

	Treatment (T)		Season (S)		T x S	
Parameter	*F*	*P*	*F*	*P*	*F*	*P*
GWC	215.68	**<.0001**	111.89	**<.0001**	20.13	**<.0001**
Soil pH	9.13	**0.002**	47.52	**<.0001**	0.92	0.509
C:N ratio	0.35	0.707	12.83	**<.0001**	1.53	0.168
TN	1.50	0.251	23.16	**<.0001**	0.75	0.648
NH_4_^+^	3.51	0.053	40.65	**<.0001**	1.46	0.191
NO_3_^−^	0.63	0.545	14.93	**<.0001**	1.91	0.075
C_org_	1.05	0.373	2.76	**0.0360**	1.76	0.104
16S gene copies	1.00	0.390	3.11	**0.0220**	0.28	0.971
18S gene copies	1.02	0.383	5.61	**0.0010**	0.35	0.942
Tree height	0.03	0.972	82.08	**<.0001**	0.64	0.740
Tree diameter	0.81	0.460	19.26	**<.0001**	2.70	**0.014**
Needle litter	0.18	0.837	14.52	**<.0001**	0.03	0.425
Photo. A_net_	75.48	**<.0001**	15.64	**<.0001**	3.73	**0.005**

Effects of treatment (T) (*n* = 3), season (S) (*n* = 5), and their interaction (*n* = 15) on soil physicochemical parameters, abundance of taxonomic markers (16S rRNA gene, 18S rRNA gene), and tree growth were tested with linear mixed effect models (lme) and displayed with the F-ratio (F) and level of significance (P). Significant results are indicated with bold numbers. GWC = gravimetric water content; TN = total nitrogen concentration; NH_4_^+^ = ammonium concentration; NO_3_^−^ = nitrate concentration; C_org_ = organic carbon concentration; and Photo. A_net_ = photosynthetic assimilation.

### Scots pine saplings response to treatments

The Scots pine saplings grew mainly from Spring-20 to Autumn-20 (subsequently referred to as growing season). The main increase in height and diameter occurred in the summer season, during which the saplings grew on average by 6.2–8.4 cm in height (Supplementary Table 2) and by 0.2–1.4 mm in diameter (Supplementary Table 2). Growth of the saplings, as measured by height and diameter, was influenced by the sampling time point (season), but we did not find consistent differences in growth among treatments (Table 1). However, saplings under the control treatment showed a larger radial growth (Supplementary Table 2), and radial growth was significantly influenced by the interaction between treatment and sampling time point (season) (*P* = .014).

As it is typical for evergreen pine trees, the amount of needle fall was highest in Autumn-20 (Supplementary Table 2). The photosynthetic assimilation was significantly affected by water limitation treatments (*P* <.0001) and sampling time points (season, *P* <.0001). Across all sampling time points (seasons), A_net_ was lower under intermediate and severe water limitation as compared to the control (Supplementary Table 2). In the control mesocosms, the greatest photosynthetic assimilation was measured in Summer-20, whereas the other mesocosms showed the largest values in Spring-20 (Supplementary Table 2).

### Soil pH, carbon, and nitrogen concentrations

Soil pH was influenced by water limitation (*P* = 0.0020) and sampling time point (season, *P* <.0001) (Table 1). The uppermost pH was measured in Winter-20, with values around 7.1 ( Supplementary Figure 1A). During the growing season of the Scots pine saplings, soil pH decreased for all irrigation treatments to 6.8–6.9 ( Supplementary Figure 1A). After the start of the irrigation treatments, soil pH remained more basic in the control mesocosms compared to the soils treated with intermediate and severe water limitation ( Supplementary Figure 1A).

Soil C_org_ and total nitrogen concentrations (TN) differed significantly among sampling time points (season) (Table 1). C_org_ tended to decrease over the experimental period, except in the soils of the control mesocosms ( Supplementary Figure 2E).

TN was similar in Winter-20 and Spring-20 but declined throughout the following sampling time points (seasons) ( Supplementary Figure 1C). TN was lower in soils treated with water-limiting conditions compared to the control; however, this difference was statistically not significant (Table   1).

Soil C:N ratios increased during the experiment (*P* <.05) (Supplementary Figure 1D). At the beginning of the irrigation treatments soil C:N ratio ranged between 16 and 20. In the following, the soil C:N ratio increased, ranging between 17 and 24 in Summer-20 and 17–29 in Autumn-20 and Winter-21.

Ammonium (NH_4_^+^) and nitrate (NO_3_^−^) concentrations decreased during the growing season of the saplings, and for all treatments, the lowest values were observed in Summer-20 (Supplementary Figure 1F and G).

### Microbial community analyses

#### Abundance of taxonomic groups

Water deficit did not affect prokaryotic and fungal abundance (Table 1, Supplementary Figure 3A, B). However, the sampling time point influenced the abundance of fungal (*P* = .0007 , Table 1) and prokaryotic (*P* = .0221, Table   1) gene copies. This was driven by the significantly larger estimated copy numbers detected in Summer-20 (Supplementary Figure 3B).

#### Soil microbial community structure

After quality filtering and taxonomic assignment, 4 166 193 16S rRNA gene sequences delineated into 19 840 ASVs, and 3993 873 ITS2 sequences delineated into 2686 ASVs were obtained across the 90 samples.

Water limitation had little effect on α-diversity (examined by observed richness, Pielou’s evenness, and Shannon diversity), but α-diversity was strongly influenced by the sampling time point (season) (Table 2, Supplementary Figure 3). Prokaryotic evenness was higher in the control treatment as compared to the water limiting treatments (Table   2). Prokaryotic α-diversity increased during Spring-20 and Summer-20 and subsequently declined during Autumn-20 and Winter-21 across all irrigation treatments. The largest variability was observed in summer ( Supplementary Figure 3C–E).

**Table 2.** Effects of irrigation treatment and sampling time point (season) on soil microbial α-diversity and β-diversity.

	⍺-Diversity									β-Diversity		
	Observed richness (S)			Pielou’s evenness (J)			Shannon diversity (H)			Bray–Curtis dissimilarity		
Prokaryotes	*R* ^2^	*F*_M_ (P)	*F*_D_(P)	*R* ^2^	*F*_M_ (P)	*F*_D_(P)	*R* ^2^	*F*_M_(P)	*F*_D_(P)	*R* ^2^	*F*_M_ (P)	*F*_D_(P)
Treatment (T)	0.01	0.8 (0.478)	0.2 (0.810)	0.02	4.3 (**0.019**)	0.5 (0.600)	0.02	2.7 (0.065)	0.5 (0.600)	0.04	2.2 **(0.001**)	11.4 (**0.001**)
Season (S)	0.66	41.6 (**0.001**)	1.1 (0.350)	0.77	81.1 (**0.001**)	1.4 (0.230)	0.76	71.5 (**0.001**)	1.0 (0.420)	0.13	3.4 (**0.001**)	9.6 (**0.001**)
T x S	0.03	1.1 (0.426)	1.1 (0.350)	0.03	1.7 (0.106)	0.7 (0.770)	0.03	1.6 (0.156)	0.84 (0.620)	0.09	1.2 (**0.003**)	2.7 (**0.002**)
	**⍺-Diversity**									**β-Diversity**		
	**Observed Richness (S)**			**Pielou’s Evenness (J)**			**Shannon Diversity (H)**			**Bray–Curtis dissimilarity**		
**Fungi**	**R^2^**	**F_M_ (P)**	**F_D_(P)**	**R^2^**	**F_M_ (P)**	**F_D_(P)**	**R^2^**	**F_M_(P)**	**F_D_(P)**	**R^2^**	**F_M_ (P)**	**F_D_(P)**
Treatment (T)	0.08	6.4 (**0.002**)	0.2 (0.870)	0.01	0.3 (0.764)	1.9 (0.140)	0.03	1.3 (0.279)	2.6 (0.066)	0.03	1.8 (**0.002**)	0.7 (0.540)
Season (S)	0.39	15.7 (**0.001**)	3.2 (**0.014**)	0.25	6.7 (**0.001**)	1.5 (0.200)	0.14	3.4 (**0.020**)	1.7 (0.170)	0.17	4.3 (**0.001**)	3.3 (**0.017**)
T × S	0.08	1.6 (0.145)	0.8 (0.680)	0.05	0.7 (0.732)	0.7 (0.790)	0.08	1.0 (0.494)	0.9 (0.560)	0.08	1.0 (0.640)	1.3 (0.280)

Effects of treatment (*n* = 3), season (*n* = 5) and their interaction (*n* = 15) on prokaryotic and fungal α-diversity and β-diversity assessed by univariate (α-diversity) and multivariate (β-diversity) permutational analysis of variance (PERMANOVA). Values indicate the F-ratio (F_M_), the level of significance (P), and the explained variance (R^2^). Significant heterogeneities of variance assessed by permutational analysis of univariate dispersion (PERMDISP) are displayed with the F-ratio (F_D_) and the level of significance (P). Significant results are indicated with bold numbers.

The observed fungal richness decreased over the course of the experiment and differed between treatments already at the beginning (Supplementary Figure 3, Table 2). From Summer-20 onward, observed richness remained largest for the severe water deficit treatment ( Supplementary Figure 3F–H). A similar response to the treatments was observed for Pielou’s evenness and Shannon diversity ( Supplementary Figure 3F–H). However, statistical tests indicated that the sampling time point (season) only significantly affected Pielou’s evenness and Shannon diversity (Table   2).

Water limitation altered prokaryotic (*P* < .001, Table 2) and fungal (*P* = .002, Table 2) β-diversity, explaining 3% and 4% of the variance, respectively (Table 2). In comparison, effects of the sampling time point (season) on β-diversity were stronger, explaining 13%–17% of the variance, respectively (Table 2). For prokaryotes, the treatment effects depended on the sampling time point (season, *P* = .003), whereas, there was no treatment-by-time interaction for fungi.

**Figure 2.**
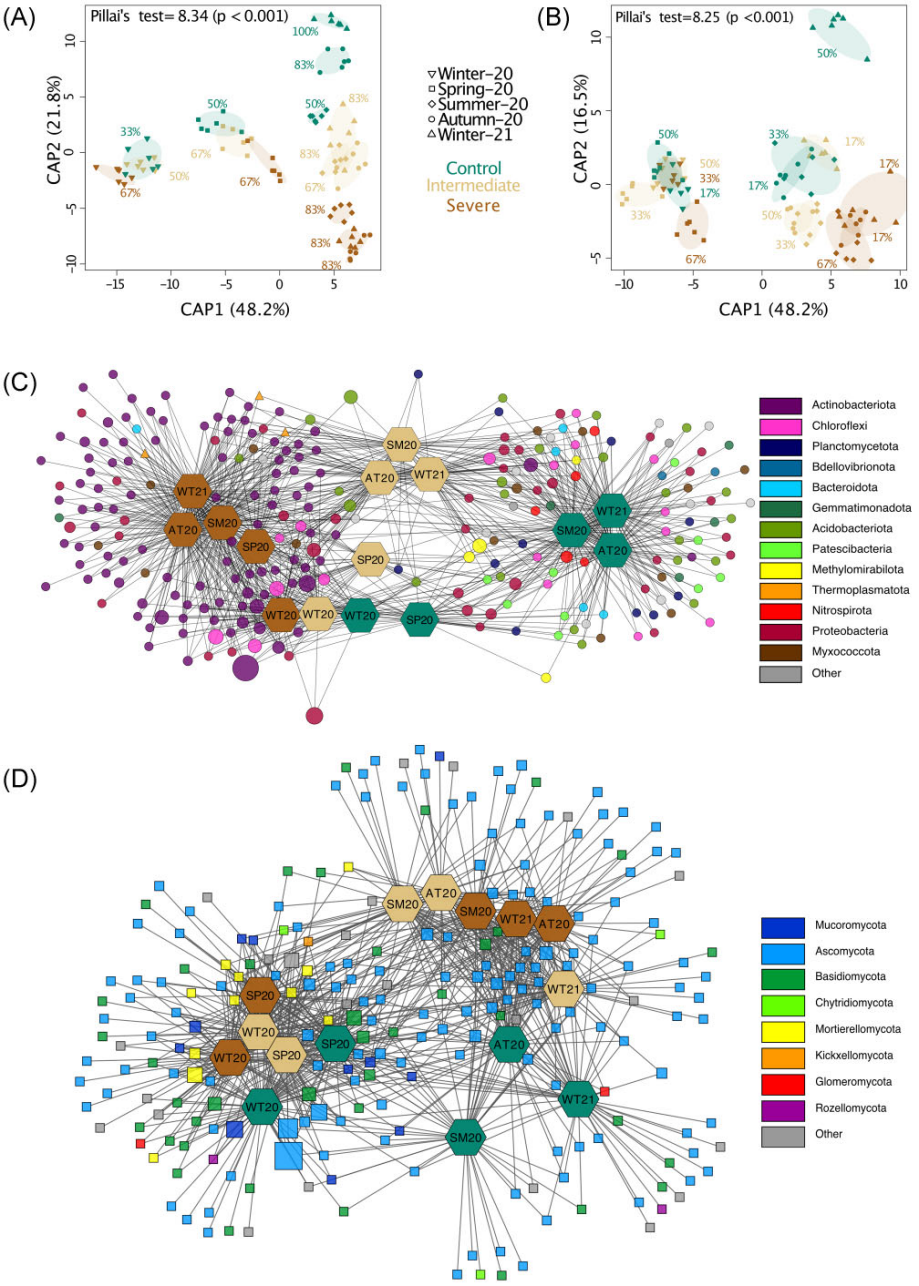
Soil microbial communities across irrigation treatments and sampling time points (seasons). Canonical analysis of principal coordinates (CAP) constraining differences in prokaryotic (A) and fungal (B) community structure by irrigation treatment and sampling time point (season). The CAP reclassification success rates providing a quantitative estimation of the degree of discrimination between the groups are provided next to each treatment and time point (season). The CAP equivalent to Pillai’s trace test (with *P*-value in brackets) indicating the overall effect size is provided at the top of the plot. (C and D) Prokaryotic and fungal bipartite association networks showing the ASV distribution (circles = prokaryotes, triangles = archaea, squares = fungi) across the different sampling time points (hexagon; seasons, WT = Winter, SP = Spring, SM = Summer, and AT = Autumn) and treatments. Node sizes are scaled by read counts (square root) and color-coded by phylum-level assignment. Edges correspond to significant associations between ASVs and samples based on indicator species analysis. The edge-weighted (weighted by ASV association strength) “Allegro Fruchterman–Reingold” algorithm was applied to the network, which clusters samples with higher connectivity ( = similar community structure).

The CAP-reclassification success rates indicated that each combination of sampling time point (season) and treatment group harbored distinct prokaryotic microbial communities (Fig. 2A), whereas reclassification success increased toward later sampling points, indicating that treatment groups become statistically more distinct. However, lower reclassification rates for fungal communities were attributed to smaller differences in the composition of these communities (Fig. 2B).

The composition of prokaryotic communities was mainly shaped by GWC, pH, TN, C_org_, and soil temperature as obtained by statistical tests (Table 3, 4, Supplementary Figure 4C). The same parameters influenced fungal communities but in a different magnitude (Table 3, 4, Supplementary Figure 4D). Overall, soil temperature —which was similar to the greenhouse air temperature— was the main driver among the measured properties during Summer-20, Autumn-20, and Winter-21 (Supplementary Figure 4C and D).

**Table 3.** Effects of measured soil parameters on the prokaryotic and fungal β-Diversity.

	β-Diversity (Bray–Curtis Dissimilarity)					
	Prokaryotes			Fungi		
	*R* ^2^	*F*	*P*	*R* ^2^	*F*	*P*
GWC	0.050	4.943	**0.001**	0.067	6.729	**0.001**
pH	0.027	2.626	**0.001**	0.018	1.783	**0.012**
C:N	0.024	2.333	**0.001**	0.022	2.244	**0.001**
C_org_	0.021	2.051	**0.001**	0.033	3.334	**0.001**
TN	0.013	1.271	0.055	0.013	1.299	0.095
NH_4_^+^	0.013	1.300	**0.047**	0.014	1.411	0.067
NO_3_^−^	0.011	1.081	0.216	0.010	0.986	0.359
Soil temperature	0.015	1.481	**0.013**	0.021	2.114	**0.003**

Effects assessed by univariate permutational analysis of variance (PERMANOVA). Values indicate the F-ratio (*F*), the level of significance (*P*), and the explained variance (*R*^2^). Significant results are indicated with bold numbers. GWC = gravimetric water content; TN = total nitrogen concentration; NH_4_^+^ = ammonium concentration; NO_3_^−^ = nitrate concentration; and C_org_ = organic carbon concentration.

**Table 4.** Effects of physicochemical soil properties on soil microbial community structure based on building a parsimonious model for variable selection with the function ordistep followed by PERMANOVA, displayed with F-ratio (*F*) and level of significance (*P*).

Prokaryotes			Fungi		
	*F*	*P*		*F*	*P*
GWC	4.921	0.001	GWC	6.688	0.001
TN	2.648	0.001	TN	3.474	0.001
pH	2.605	0.001	pH	2.076	0.002
C_org_	1.822	0.002	C_org_	2.047	0.005
Soil temp.	1.644	0.009	Soil Temp.	2.045	0.002

GWC = gravimetric water content; TN = total nitrogen concentration; and C_org_ = organic carbon concentration.

#### Taxa sensitive to water deficit

After correction for multiple testing, 749 out of 19 840 (3.8%) prokaryotic ASVs were observed to be significantly (PERMANOVA; *q* <.05) influenced by water deficit. Out of these, 90 ASVs could be assigned at the genus level. 257 ASVs with a strong (F-ratio ≥ 10) and significant (*q* <.05) change in relative abundance were selected for the indicator species analyses and subsequent construction of the bipartite association network. Since only 13 out of 2686 fungal ASVs responded significantly to water deficit (*q* <.05), a less strict threshold of *q* <.1 was applied, resulting in 130 fungal ASVs (4.8% of total ASVs) that were used for indicator species analysis and bipartite network construction, with 21 ASVs assigned to genus level. Significant changes in relative abundances were also statistically evaluated at all taxonomic levels by aggregating the data based on summarized ASV counts from genus to phylum level.

**Figure 3.**
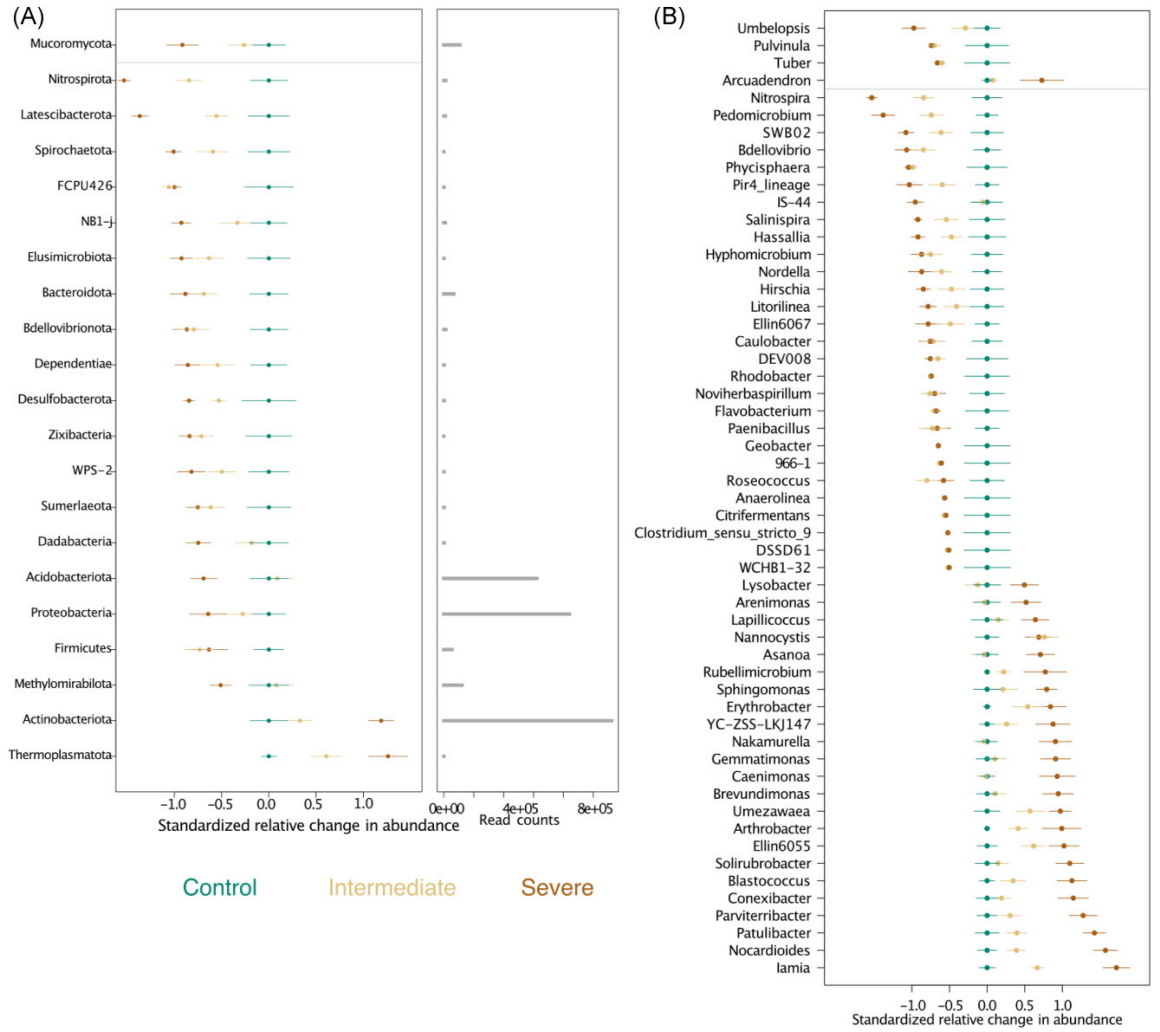
Significant (*q* < .05) changes in relative abundance of phyla and genera under water stress. Relative change (z-transformed) in abundance of fungal and bacterial phyla (A) or genera (B) under intermediate and severe water deficit compared to the control treatment. The first vertical panel represents the relative change in abundance from the overall mean, including the average change (circles) and the corresponding standard error (horizontal lines)—only phyla (A) and genera (B) with significant change in abundance are displayed. The second vertical panel of (A) represents the relative abundance of the phyla (horizontal grey bars) as obtained by read counts.

A decrease in relative abundance under intermediate and severe water deficit compared to the control was observed for most of the responsive (*q* <.05) prokaryotic phyla (Fig. 3A). Treatment-sensitive ASVs were broadly distributed across the taxonomic hierarchy, revealing substantial response heterogeneity within individual phyla.

While, the overall abundance of the phylum Proteobacteria declined under water deficit, this was heterogenous at the genus level. The genera *Roseococcus, Caulobacter, Hyphomicrobium, Pedomicrobium, Rhodobacter, Coxiella*, and *Massilia* decreased in relative abundance under water deficit (Fig. 3B). On the contrary, genera such as *Erythrobacter, Novosphingobium, Sphingomonas, Arenimonas*, and *Lysobacter* increased under water deficit (Fig. 3B). The phyla Acidobacteriota and Methylomirabilota increased in relative abundance compared to the control, but only under intermediate and not under severe water deficit (Fig. 3A).

Only the relative abundance of Actinobacteriota and the archaeal phylum Thermoplasmatota significantly (*q* <.05) increased under severe water deficit compared to the control treatment (Fig. 3A), with a strong enrichment of the Actinobacteriota also clearly visible at the ASV level (Fig. 2C). Salient examples of bacterial genera belonging to the Actinobacteriota with ASVs increasing under severe water deficit included *Blasterococcus, Nakamurella, Nocardioides, Actinophytocola, Patulibacter*, and *Solirubrobacter* (Fig. 3B). No assignment at the genus level could be obtained for the archaeal phylum Thermoplasmatota.

Mucoromycota was the only fungal phylum that changed due to the irrigation treatment and declined in relative abundance under severe and intermediate water deficits compared to control conditions (Fig. 3A). Examples of fungal genera belonging to the Mucoromycota phylum with ASVs decreasing under intermediate and severe water deficit included *Absidia* and *Umbelopsis* (Fig. 3B).

Fungal phyla that showed a stable abundance under water limitation included Basidiomycota and Mortierellomycota (Fig. 2D), although these phyla showed different responses to water limitation at the genus level (Fig. 3B). Salient examples of fungal genera belonging to these phyla included *Amphinema, Cystobasidium, Paratritirachium, Udeniozyma*, and *Mortierella* for the Mortierellomycota (Fig. 3B). An overall trend toward an increased relative abundance of ASVs under intermediate and severe water deficit was observed for the fungal phylum Ascomycota (Fig. 2D). Since the phylum Ascomycota was dominant in all samples, this increase was, however, contrasting at the genus level, e.g. with the genera *Niesslia, Trichophaea*, and *Helicodendron* increasing and *Curvularia, Pulvinula, Staphylotrichum, Tuber*, and *Stachybotrys* decreasing under water deficit (Fig. 3B).

## Discussion

### Effect of soil water content on soil microbial communities

One year of experimental soil water limitation did not alter the total abundance of prokaryotes and fungi (Supplementary Figure 3A and B). Nevertheless, water limitation shaped the composition of the soil microbiome of the Scots pine mesocosms (Fig. 2, 3), confirming that water deficit promotes distinct microbial communities. This finding corroborates results from long-term field observations indicating that changes in forest irrigation patterns significantly altered microbial communities due to different abilities to cope with water scarcity and modified substrate availabilities (Hartmann et al. 2017).

We observed negative responses to water limitation for most of the responsive prokaryotic phyla but only for one fungal phylum. The decrease in relative abundance of sensitive phyla was mainly gradual and more substantial under severe water limitation (Fig. 3A). While the fungal α-diversity remained higher under water limitation, the opposite pattern was observed for prokaryotic α-diversity (Supplementary Figure 3). Fungal communities appeared to have a greater tolerance toward water limitation than prokaryotic communities (Figs. 2B, 2D, 3A, 3B). This observation aligns with a study by de Vries et al. (2018), who observed a greater resistance of fungal networks compared to bacterial networks under drought. Contrary, other studies found a greater tolerance of bacterial communities under simulated global change (Martiny et al. 2017) or minor effects of rainfall exclusion on community compositions (Ren et al. 2018). Moreover, Sayer et al. (2017) reported shifts in soil fungal and bacterial community structure in response to long-term drought and a substantial loss of fungal taxa. Since the results presented in this study have been generated over one year, more significant changes in fungal community composition might only become evident at longer time scales. However, some first changes in the composition of fungal communities could already be detected (Fig. 3A and  B).

The greater tolerance of fungal communities to ongoing water limitation (Figs. 2B, 2D, 3A, 3B) is likely explained by the ability of fungi to create large hyphal networks, which allows them to enter water-filled small soil pores and, thereby, maintain nutrient and water uptake over long distances (Allen 2007, Joergensen and Wichern 2008). Uncertainty remains whether the tolerance to water deficit indicates an adaptation of soil-inhabiting fungi or rather the presence of abundant dormant propagules, which can withstand adverse environmental conditions and regain metabolic activity once favorable conditions return (Lennon and Jones 2011, Barnard et al. 2013, Meisner et al. 2018). The soil used in the study was collected from a xeric forest and stored for some months before the start of the experiment. Hence, the observed tolerance of fungal communities might be related to the presence of abundant dormant fungal taxa, which can withstand unfavorable environmental conditions and regain metabolic activity upon rewetting (Lennon and Jones 2011, Barnard et al. 2013, Meisner et al. 2018).

### Effect of seasonality on soil microbial communities

The seasonal sampling time points profoundly governed the variability in the composition of both fungal and prokaryotic communities (13%, respectively, 17%, Table 2). Seasonality is well known to affect the activity and composition of microbial communities through alterations in soil moisture and temperatures (Moore-Kucera and Dick 2008, Cruz-Martínez et al. 2009, Kuffner et al. 2012, Vořiškova et al. 2014, Žifčáková et al. 2017). Soil temperature is essential to microbial growth, metabolism, and physiology (Schimel et al. 2007, Madigan et al. 2018). However, high soil temperatures also create unfavorable conditions setting inhibitory boundaries for microbial growth (Sheik et al. 2011). In our experiment, although soil water content was carefully controlled with volumetric water sensors in each mesocosm, some confounding effects of seasonal changes in soil moisture could not be avoided (Fig. 1). Higher soil evapotranspiration rates primarily reduced soil water contents during summer due to temperature increases (Fig. 1). The concurrent increment in soil temperatures and lower soil moisture levels during the growing season likely stimulated the proliferation of communities tolerant to higher temperatures and dehydration and might have led to the observed strong effect of the sampling time point (season).

The observed effect of seasonality on soil microbial communities was also likely related to the periodic differences in plant growth and litter production (Myers et al. 2001, Wardle et al. 2004, Högberg 2006). Trees typically provide readily available C substrates during the growing season for microbes stimulating their activity and growth (Hobbie 2006). These seasonal differences in C and nutrient availabilities could thereby explain the strong influence of the seasonal time point on the microbial communities. Although water limitation affected the photosynthetic C assimilation of the saplings in our study, aboveground growth and needle litter production remained sustained during the growing season (Supplementary Table 2). This pattern likely led to the peak in prokaryotic α-diversity during the growing period of the Scots pine saplings (Supplementary Figure 3C, D, and E) and might have favored the higher abundance of microbes in summer as estimated by measuring 16S and 18S rRNA gene copy numbers (Supplementary Figure 3A and B). Moreover, the strong influence of the sampling time point could be related to successional changes in tree development after the establishment of the mesocosms.

### Desiccation-tolerant microbial taxa

Soil microbial communities were dominated by phyla commonly observed in soil, including Proteobacteria, Actinobacteriota, and Acidobacteriota, as well as Basidiomycota and Ascomycota (Fig. 2C, D). The fungal phyla Ascomycota and Basidiomycota appeared to tolerate water limitation, resulting in a dominance of these phyla in all our samples (Fig. 2D). This finding corroborates the results published by Hartmann et al. (2017), who observed a stable abundance of Ascomycota and an increase of Basidiomycota under long-term dry conditions in a Scots pine forest.

The bacterial phylum Actinobacteriota significantly increased in relative abundance with increasing soil water limitation (Fig. 3A). Observations by Hartmann et al. (2017), coincide with our results as the abundance of Actinobacteriota increased in water-limited Scots pine forest soils. In our experiment, the abundance of aerobic genera commonly found in sandy and rocky soils (França et al. 2016, Sghaier et al. 2016), like *Blasterococcus, Nakamurella, Actinophytocola*, and *Nocardioides*, increased with increasing water deficit (Fig. 3B). Furthermore, the genera *Patulibacter* and *Solirubrobacter*, which are thermophilic and often associated with arid soils (Acosta-Martínez et al. 2014, Albuquerque et al. 2014, Bastida et al. 2017, Tóth et al. 2017), also increased under water deficit (Fig. 3B). As a result, the above-mentioned taxa might be tolerant to water deficits and could serve as indicators of soils with low soil water content.

The archaeal phylum Thermoplasmatota increased in relative abundance under severe water deficit (Fig. 3A). Thermoplasmatota seem tolerant toward environmental stress because they are found in hot and acidic environments (Tripathi et al. 2015). Thus, the increase of Thermoplasmatota in the mesocosms affected by severe water deficit potentially contributed to prokaryotic communities, which are more tolerant toward environmental stress.

Contrary to Hartmann et al. (2017), who observed an increase of Acidobacteriota under dry conditions, in our study, Acidobacteriota were not affected by intermediate water limitation (Fig. 3A). This differing result might be explained by the fact that the severity of water limitation was more profound in our study than in the field site studied by Hartmann et al. (2017). However, the observed sustained abundance of Acidobacteriota under intermediate water-limiting conditions suggests a tolerance threshold of Acidobacteriota toward moderately arid conditions. Acidobacteriota are ubiquitous, desiccation tolerant, and adapted to nutrient-limited environments (Kielak et al. 2016a), as they are commonly found to be abundant in environments characterized by low resource availability (Fierer et al. 2007, Koyama et al. 2014). This adaptability could explain the observed resistance toward intermediate water-limiting conditions. Moreover, it has been observed that Acidobacteriota produce extracellular polysaccharides that can create moist micro-niches (Kielak et al. 2016a), which might benefit other bacteria during episodes of dry conditions.

Barnard et al. (2013), also found a relative increase of Actinobacteriota and a decrease of Acidobacteriota with summer dry-down and concluded that these contrasting drought-associated changes in abundance might reflect different desiccation-related bacterial life strategies. This reasoning could also serve as an explanation for the pattern observed in our study.

### Changes in microbial communities related to altered soil properties

We observed a decrease in soil pH under water limitation (Supplementary Figure 1A), which could be explained by an accumulation of cations due to reduced leaching and plant uptake. A significant relationship between soil pH and community structure of both prokaryotic and fungal communities was observed in our mesocosms (Tables 3, 4). However, the relationship with pH was lower for fungal as compared to prokaryotic communities, as also observed in previous studies (Rousk et al. 2010, Siles and Margesin 2016). In our study, fungi were more sensitive to variations in other soil physiochemical properties as compared to variations in soil acidity (Table 3).

Changes in soil pH can directly affect soil microbes by altering their competitive fitness or impairing the growth of individual taxa that cannot survive when soil pH falls outside a certain range (Lauber et al. 2009, Madigan et al. 2018). Therefore, the decreasing pH observed in our study might have imposed considerable stress on microbes that was only tolerated by certain taxa. For example, we found an increase of Actinobacteriota associated with a decrease in soil pH.

However, the strong influence of soil pH on soil microorganisms can also be explained through indirect effects (Lammel et al. 2018). For example, soil pH shapes several soil characteristics important for microbes, such as e.g. solubility of ions and nutrients and salinity (Rousk et al. 2010, Brady and Weil 2013, Zhalnina et al. 2015, Madigan et al. 2018, Solly et al. 2020). In turn, these indirect effects of soil pH may also drive the observed changes in community composition and thus could explain the strong influence of soil pH on microbial communities in our study.

C_org_ also significantly influenced the composition of microbial communities (Table 4, Supplementary Figure 4C, 4D). Organic C is known to be the primary energy substrate for microbes under oxic conditions; thereby, a reduction of C_org_ can rapidly alter microbial communities toward oligotrophic microbial groups, which can survive under limited C resource availability (Aldén et al. 2001, Drenovsky et al. 2004, Soong et al. 2020). Therefore, in our study, C_org_ potentially contributed to a proliferation of competitive taxa able to survive under a low availability of organic C and specialized in the degradation of more recalcitrant C compounds.

During the experiment, we observed an increase in the C:N ratio related to a decline in TN (Supplementary Figure 1C, D). Increases in soil C:N ratios are often used as a proxy indicating an accumulation of complex organic compounds —such as lignin— which favors microbes with the ability to decompose recalcitrant structures (Baldrian et al. 2012, Van der Wal et al. 2013, Lindahl and Tunlid 2015, Žifčáková et al. 2017). We detected a lower abundance of Acidobacteriota in samples with a greater C:N ratio, which could be related to their adaptation to oligotrophic conditions (Naether et al. 2012). Hartmann et al. (2017) hypothesized that taxa, which can metabolize more recalcitrant compounds have an advantage when the input of easily degradable plant resources is limited. In our experiment, complex compounds likely accumulated in the mesocosms under the reduced and severe water limitation treatments, favoring the abundance of saprotrophic taxa. With higher C:N ratios, Basidiomycota, Ascomycota, and Actinobacteriota increased in abundance (Fig. 3). Many genera of the Actinobacteriota are saprophytic and were shown to be highly resistant to desiccation and C starvation (Ventura et al. 2007, Rosenberg et al. 2014, Mohammadipanah and Wink 2016). Also, the dominant fungal phylum Basidiomycota comprises —next to other functional groups as symbionts —many saprotrophic genera (Tedersoo et al. 2010), which degrade complex compounds, including cellulose and lignin (Baldrian 2008). For instance, the saprotrophic genus *Paratritirachium* (Tedersoo et al. 2014), significantly increased in abundance with water limitation (Fig. 3B). Many genera belonging to the highly abundant phylum Ascomycota are further described as saprotrophs (Tedersoo et al. 2010, Voriskova and Baldrian 2013). For example, we found a significant increase of the saprotrophic genera *Niesslia* (Tedersoo et al. 2014) and *Helicodendron* (Tedersoo et al. 2014) (Fig. 3B). Next to being symbionts, most of the Ascomycota and Basidiomycota possess the capability to degrade cellulose (De Boer et al. 2005). Consequently, some saprophytic populations might suffer from resource competition during decomposition (Sollins et al. 2007).

### Relationship between microbial communities and Scots pine saplings

More extreme episodes of drought, which lead to soil moisture levels below the wilting point, are a major predisposing risk for tree mortality events (McDowell et al. 2022). To ensure that the Scots pines remained vital, the saplings received a minimum of water in our experiment. The photosynthetic assimilation and aboveground growth of the Scots pine saplings were altered by severe water limitation. Nevertheless, we observed no direct effect of the studied aboveground plant parameters on microbial communities. This study did not focus on belowground plant traits because we could not destructively sample the mesocosms but collected different soil cores from the same soil system while the experiment was still ongoing. Nevertheless, our findings reveal significant changes in the abundance of symbiotic taxa associated with changing physicochemical soil properties under drier conditions with potential consequences for developing young trees.

Our results indicate that the abundance of the proteobacterial family Rhizobiales significantly declined under intermediate and severe water deficits compared to the control (Fig. 3B). Rhizobiales are well-studied associates of plants, and they commonly exert beneficial functions for their hosts, e.g. through N-fixation (Delmotte et al. 2009). Thus, their decline does corroborate our hypothesis of a decrease of symbionts under water stress. Furthermore, Proteobacteria were shown to be sensitive to short-term drought scenarios (Bouskill et al. 2013, Chodak et al. 2015), supporting our finding. Moreover, our observation is in line with the results from a forest field site where Hartmann et al. (2017) found an increase of the phylum Proteobacteria in plots under irrigation, presumably linked to their copiotrophic lifestyle, which is found in environments with rich plant-C inputs (Fierer et al. 2007). This observation could also explain the better plant performance (growth and photosynthesis) under the control treatment in which the relationship with symbionts might have sustained the saplings. Furthermore, we found a decrease of diazotrophic bacteria such as the endophytic genus *Paenibacillus* (Fig. 3B), which is known to improve the nutrient status of plants through phosphate solubilization and N-fixation under dry and nutrient-poor conditions (Bal et al. 2012, Puri et al. 2018), additionally supporting our expectation of a decline of symbionts. The observed decrease of N-fixing bacteria may have consequences for plant nitrogen availability and might partially explain the reduced soil ammonium concentrations under drier conditions.

Also, the fungal phyla Basidiomycota and Ascomycota encompass many symbiotic taxa (Tedersoo et al. 2010, Voriskova and Baldrian 2013), of which we observed a decrease and thus supporting our hypothesis. For example, the family Tuberaceae (Ascomycota), known as truffles, forms symbiotic associations with plants (Trappe et al. 2009), and was found to decrease in mesocosm with water limitation. Further salient examples of putative EcM declining in abundance under water deficit included *Amphinema* (Basiodmycota) and *Pulvinula* (Ascomycota) (Fig. 3B) (Rinaldi et al. 2008, Tedersoo et al. 2010).

In our study, the phylum Mucoromycota was the only fungal phylum significantly affected by water limitation (Fig. 3A). As opportunists, Mucoromycota thrive on readily available C-sources such as monosaccharides from root exudations (Dix and Webster 1995). These C-sources might be limited under water deficit as fresh and easily degradable C inputs of host plants might be impaired due to reduced photosynthetic activity under low soil moisture. This lack of easily degradable C inputs could explain the observed significant decrease of this phylum in our study and matches with an increase of this phylum in irrigated soils of a Scots pine forest observed by Hartmann et al. (2017). Moreover, it is proposed that some families of the fungal phylum Mucoromycota are involved in symbiosis with plants (Field et al. 2015). This finding suggests that Mucoromycota might form mutualistic associations, indicating a functional overlap with EcM fungi (van der Heijden et al. 2015), further supporting our hypothesis of reduced symbionts under water stress.

Overall, our findings indicate that soil water limitation in Scots pine forest soils will likely lead to a decrease in the relative abundance of N-fixing bacteria and symbiotic microbial taxa of trees, with cascading consequences on plant nutrition and forest health.

## Concluding Remarks

Our study revealed that under water limitation, soil microbial communities in Scots pine mesocosms are rather shaped by alterations in soil properties such as pH and C:N ratio than by the direct influence of measured sapling growth parameters. Our findings further demonstrate a strong effect of seasonal changes in soil temperature and soil water content and a gradual response of most sensitive phyla to contrasting levels of water limitation. These results contribute to our understanding of how soil microbial communities adapt to different thresholds of water limitation and support the current view that soil prokaryotes are generally more sensitive to water limitation than soil fungi.

The presented results indicate that water limitation promotes the proliferation of microbial groups tolerant of environmental stress. Shifts in microbial community compositions induced by water limitation led to an accumulation of desiccation-tolerant taxa, potentially altering critical functions provided by the forest soil microbiome, such as nutrient cycling. Our data further suggest a shift related to the potential lifestyle of microbes as we recognized a decrease of symbiotic and an increase of saprotrophic taxa, potentially affecting water and nutrient availability for young trees. Overall, our findings contribute to a better understanding of how ecosystem functions mediated by soil microorganisms may be impaired in forests affected by drier conditions.

## Supplementary Material

fiad051_Supplemental_Files

## Data Availability

The raw sequencing reads of this study have been deposited in the European Nucleotide Archive (ENA) at EMBL-EBI under accession number PRJEB53192 (https://www.ebi.ac.uk/ena/browser/view/PRJEB53192).
