# Supplementary material for: Contrasting sensitivity of soil bacterial and fungal community composition to one year of water limitation in Scots pine mesocosms
Source: FEMS Microbiol Ecol. 2023 May 15;99(6):fiad051. doi: 10.1093/femsec/fiad051 (PMC10243993; doi:10.1093/femsec/fiad051)
Supplement: fiad051_Supplemental_Files [file fiad051_supplemental_files.zip › Supp_data _revised2.docx]

**Supplementary Table 1: Overview of the greenhouse conditions.** Five sampling dates across the seasons Winter-20, Spring-20, Summer-20, Autumn-20, and Winter-21. Mean and standard deviation of the greenhouse temperature (Temp. GH), the greenhouse humidity (Humidity GH), and the soil temperature (Temp. Soil) for every season.

| **Season** | **Autumn-19** | | **Winter-20** | **Spring-20** | **Summer-20** | **Autumn-20** | **Winter-21** |
| --- | --- | --- | --- | --- | --- | --- | --- |
| **Sampling Date** | | 25.09.2019 | 13.01.2020 | 11.05.2020 | 27.07.2020 | 19.10.2020 | 25.01.2021 |
| **Temp. GH [°C]** | | 11.1 ± 0.1 | 10.3 ± 0.2 | 16.5 ± 0.1 | 21.5 ± 0.1 | 15.9 ± 0.1 | 10.3 ± 0.1 |
| **Temp. Soil [°C]** | | 14.7 ± 0.2 | 9.1 ± 0.1 | 17.5 ± 0.1 | 21.9 ± 0.1 | 14.5 ± 0.1 | 9.4 ± 0.1 |
| **Humidity GH [%]** | | 71.1 ± 4.6 | 52.9 ± 1.7 | 46.9 ± 0.3 | 64.3 ± 0.8 | 65.9 ± 0.5 | 51.0 ± 0.7 |

**Supplementary Table 2: Seasonal tree/stem growth, photosynthetic capacity, and needle litter fall**. Mean height and diameter with standard error measured for all 18 saplings in Winter-20 season. Mean increase in height compared to previous season (seasonal tree growth) and seasonal increase in diameter (seasonal stem growth) with standard error. Mean photosynthetic capacity (A_net_) for each treatment and standard error measured each season after start of the irrigation treatments. Mean and standard error of the dry needle litter amount collected during each season.

| **Season** | **Winter-20** | **Spring-20** | **Summer-20** | **Autumn-20** | **Winter-21** |
| --- | --- | --- | --- | --- | --- |
| **Tree height [cm]** | | **Seasonal tree growth [cm]** | | | |
| Control | 61.0 ± 2.9 | 1.9 ± 0.5 | 8.4 ± 0.5 | 1.0 ± 0.4 | 0.8 ± 0.3 |
| Intermediate | 62.9 ± 1.7 | 1.7 ± 0.6 | 6.2 ± 2.2 | 1.3 ± 0.5 | 0.7 ± 0.2 |
| Severe | 62.4 ± 2.6 | 2.5 ± 1.1 | 6.8 ± 1.4 | 0.8 ± 0.5 | 0.0 ± 0.1 |
| **Tree diameter [mm]** | | **Seasonal stem growth [mm]** | | | |
| Control | 21.6 ± 1.8 | 0.5 ± 0.8 | 1.1 ± 0.5 | 1.4 ± 0.4 | 0.1 ± 0.4 |
| Intermediate | 20.3 ± 1.5 | 0.0 ± 0.6 | 1.4 ± 0.3 | 0.3 ± 0.3 | 0.0 ± 0.2 |
| Severe | 23.0 ± 1.1 | 0.4 ± 0.2 | 0.2 ± 0.2 | 0.1 ± 0.3 | 0.1 ± 0.4 |
| **Photosynthesis (A_net_) [µmol m^-2^ s^-1^]** | | | | | |
| Control |  | 11.4 ± 0.5 | 12.6 ± 1.5 | 7.0 ± 1.0 | 7.8 ± 0.7 |
| Intermediate | | 10.5 ± 0.8 | 8.2 ± 1.4 | 4.4 ± 1.0 | 5.9 ± 0.7 |
| Severe |  | 4.4 ± 0.4 | 1.0 ± 0.4 | 0.7 ± 0.7 | 3.5 ± 0.7 |
| **Dry needle litter [g]** | | | | | |
| Control | 0.6 ± 0.2 | 1.7 ± 0.3 | 4.3 ± 2.0 | 7.8 ± 3.6 | 1.1 ± 0.4 |
| Intermediate | 1.7 ± 0.9 | 2.0 ± 0.4 | 4.0 ± 1.1 | 9.7 ± 3.3 | 1.4 ± 0.5 |
| Severe | 1.0 ± 0.5 | 1.7 ± 0.5 | 2.0 ± 0.8 | 6.0 ± 4.5 | 0.5 ± 0.2 |

**Supplementary Figure 1: Changes in soil properties across sampling time points (seasons).** Mean soil pH (A), mean total carbon concentrations in % (B), mean total nitrogen concentrations in % (C), mean C:N ratio (D), mean organic carbon concentrations in % (E), mean ammonium concentrations per gram dry weight soil (F), mean soil nitrate concentrations per gram dry weight soil (G) for each treatment group across all sampling time points (seasons), displayed with standard error. Small letters indicate significant differences between groups as obtained by estimated marginal means of linear mixed effect models. Capital letters indicate significant differences between sampling time points (seasons) as obtained by estimated marginal means of linear mixed effect models.

**Supplementary Figure 2: Rarefaction curves and read counts across the 90 samples for prokaryotes (A, C) and fungi (B, D)**.

**Supplementary Figure 3: Abundance and α-diversity of prokaryotic and fungal communities.** Mean estimated copies of the prokaryotic 16S rRNA gene (A) and the fungal 18S rRNA gene (B) assessed for each treatment across five sampling time points (seasons) with qPCR assays. Error bars indicate the standard error and capital letters indicate significant differences between sampling time points (seasons) as obtained by estimated marginal means of linear mixed effect models. The mean (± standard error) α-diversity examined by observed richness, Pielou’s evenness, and Shannon diversity for each treatment group across five sampling time points (seasons) for prokaryotes (C-E) and fungi (F-H). Significant differences between groups are indicated with small letters.

**Supplementary Figure 4: Soil microbial community structure and influence of soil properties.** Principal coordinate analysis (PCO) ordinations of prokaryotic (A) and fungal (B) communities based on Bray-Curtis dissimilarities calculated from ASV abundances. Distance-based redundancy analysis (dbRDA) showing the significant (p < 0.05) relationship between prokaryotic (C) and fungal (D) community structure with the measured physicochemical soil properties.
